# Blocking the PAH2 domain of Sin3A inhibits tumorigenesis and confers retinoid sensitivity in triple negative breast cancer

**DOI:** 10.18632/oncotarget.9905

**Published:** 2016-06-07

**Authors:** Nidhi Bansal, Almudena Bosch, Boris Leibovitch, Lutecia Pereira, Elena Cubedo, Jianshi Yu, Keely Pierzchalski, Jace W. Jones, Melissa Fishel, Maureen Kane, Arthur Zelent, Samuel Waxman, Eduardo Farias

**Affiliations:** ^1^ The Tisch Cancer Institute, Icahn School of Medicine at Mount Sinai, New York, NY, USA; ^2^ Division of Hemato-Oncology, Department of Medicine, Sylvester Comprehensive Cancer Center, Miller School of Medicine, University of Miami, Miami, FL, USA; ^3^ Department of Pharmaceutical Sciences, University of Maryland, School of Pharmacy, Baltimore, MD, USA

**Keywords:** Sin3A, SID decoys, triple negative breast cancer, retinoids, metastases

## Abstract

Triple negative breast cancer (TNBC) frequently relapses locally, regionally or as systemic metastases. Development of targeted therapy that offers significant survival benefit in TNBC is an unmet clinical need. We have previously reported that blocking interactions between PAH2 domain of chromatin regulator Sin3A and the Sin3 interaction domain (SID) containing proteins by SID decoys result in EMT reversal, and re-expression of genes associated with differentiation. Here we report a novel and therapeutically relevant combinatorial use of SID decoys. SID decoys activate RARα/β pathways that are enhanced in combination with RARα-selective agonist AM80 to induce morphogenesis and inhibit tumorsphere formation. These findings correlate with inhibition of mammary hyperplasia and a significant increase in tumor-free survival in MMTV-Myc oncomice treated with a small molecule mimetic of SID (C16). Further, in two well-established mouse TNBC models we show that treatment with C16-AM80 combination has marked anti-tumor effects, prevents lung metastases and seeding of tumor cells to bone marrow. This correlated to a remarkable 100% increase in disease-free survival with a possibility of “cure” in mice bearing a TNBC-like tumor. Targeting Sin3A by C16 alone or in combination with AM80 may thus be a promising adjuvant therapy for treating or preventing metastatic TNBC.

## INTRODUCTION

Triple Negative Breast Cancer (TNBC), representing 15–20% of all breast cancers, is an aggressive subtype with high rate of relapse, chemoresistance and decreased overall survival. Characterized as both Estrogen and Progesterone receptor negative, TNBC also lacks overexpression of the HER2 receptor [1]. This, along with molecular heterogeneity and paucity of clinically validated drug targets, contributes to the poor prognosis of TNBC. TNBC patients often receive conventional chemotherapy that has shown modest survival benefit [2, 3]. To increase the overall survival and reduce the relapse rate in TNBC patients, there is an urgent need to identify novel targeted therapeutics. We have previously reported that the chromatin remodeling protein Sin3A is a potential drug target in TNBC [4, 5]. We have developed peptides and small molecule mimetic inhibitors (SMIs) that block protein-protein interactions between the PAH2 domain of Sin3A and Sin3-interaction domain (SID) containing chromatin-associated factors like MAD1 and PF1 [6, 7]. This interference results in basal to luminal differentiation by epigenetic modulation and transcriptional repression of genes that promote epithelial to mesenchymal transition, invasion and stemness [4, 6, 7]. The potential of SID decoys is further accentuated by their ability to modulate therapeutically targetable signaling pathways, [6, 7] opening the avenue for combinatorial therapies that target specific vulnerabilities and are fundamental for an improved clinical outcome.

One of the pathways that is inactivated in many breast cancers, including TNBCs, is the retinoid pathway [8–10]. Retinoids are a family of endogenous and synthetic signaling molecules related to Vitamin A that control diverse cellular functions including, cell proliferation, differentiation and organ development [11]. Retinoids bind to two different families of nuclear retinoic acid (RA) receptors, the retinoic acid receptors (RARs) and the retinoid X receptors (RXRs) each with three subtypes (α, β, γ). All-trans-retinoic acid (atRA), a biologically active form of Vitamin A and a cytodifferentiating agent is being successfully used for the treatment of acute promyelocytic leukemia (APL) in which RARα is fused to PML [12–14]. There has been considerable interest in the use of retinoids in breast cancer treatment but the clinical results of such trials have been disappointing [15]. Factors that contribute to the failure of these clinical trials include lack of molecular determinants to predict retinoid sensitivity, expression of enzymes that metabolize retinoids, wrong choice of retinoids and epigenetic silencing of target RARs [15–17] (RARα and RARβ) by promoter methylation [15, 17–20]. Since ligand-activated RARs trans-activate complex gene networks to promote differentiation and other anti-tumor effects [11], their reduced expression prevents endogenous and exogenous retinoids from proper function. This problem can possibly be overcome by employing strategies to increase expression of RARα/β. Here we report the ability of SID decoys to activate RARα/β receptor specific pathways and their potential use in combination with RARα-selective agonist AM80 as a novel adjuvant therapy to treat metastatic TNBC.

## RESULTS

### SID peptide increases expression of RAR*β2*, endogenous retinoic acid levels and activates RAR-target promoters

**Figure 1:**
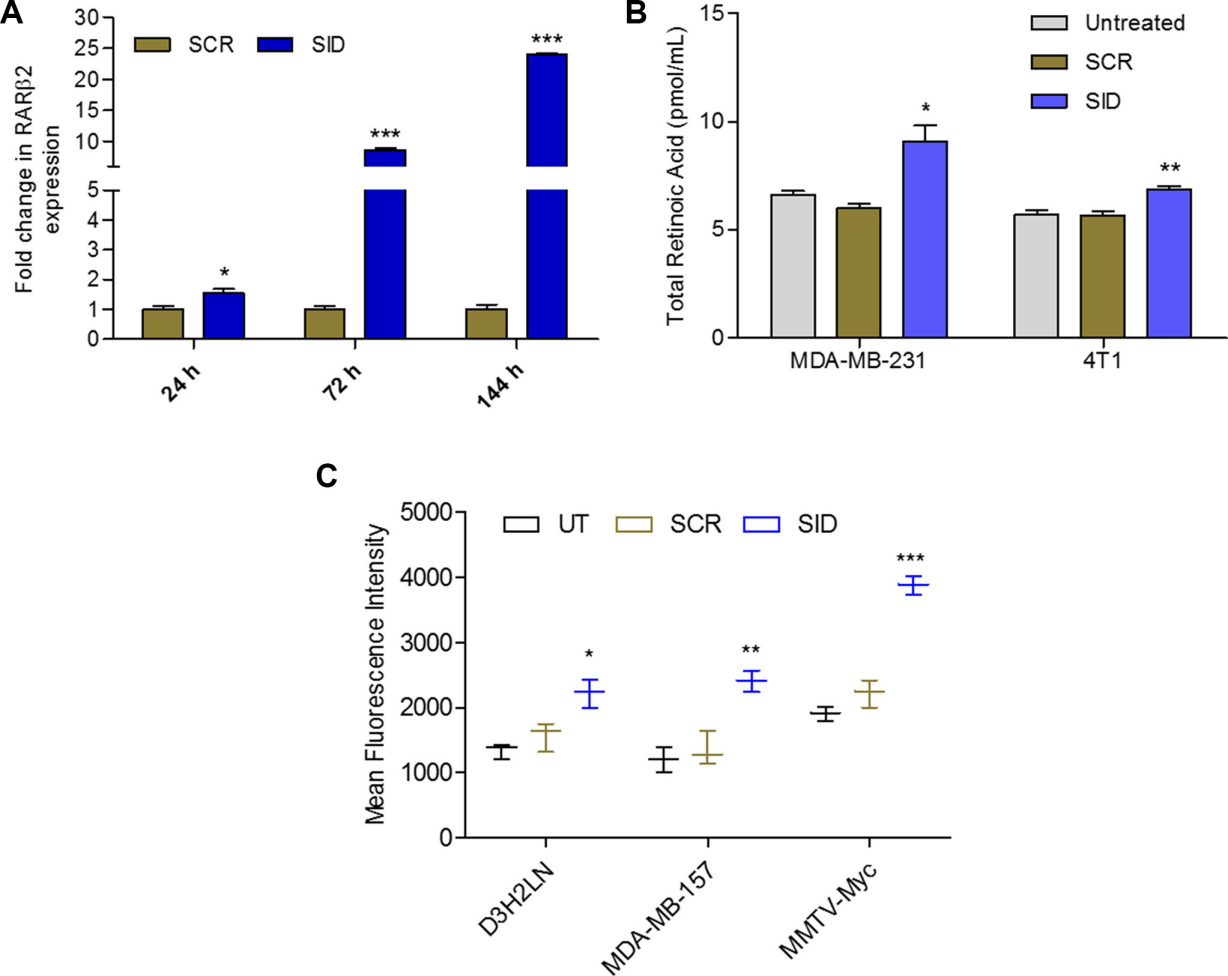
SID peptide increases expression of *RARβ2*, endogenous retinoic acid levels and activates RARE-driven promoter (**A**) qRT PCR for expression of *RARβ2* in MDA-MB-231 cells treated with 2.5 μM SID peptide for 24 h, 72 h and 144 h. Error bars represent mean ± SD (*n* = 3). SCR vs SID, *p = 0.0110 (24 h); ***p < 0.0001 (72 h and 144 h), unpaired *t*-test. (**B**) Endogenous retinoic acid levels in MDA-MB-231 and 4T1 cells, untreated or treated with 2.5 μM SCR or SID peptides. Error bars represent mean ± SD (*n* = 3). SCR versus SID, *p = 0.0145 (MDA-MB-231); **p = 0.0051 (4T1); unpaired *t*-test. (**C**) Expression of RARE-driven GFP reporter in MDA-MB-231 variant D3H2LN, MDA-MB-157 and MMTV-Myc cells treated with 2.5 μM SCR or SID peptides. Error bars represent mean ± SD (*n* = 3). SCR vs SID, *p = 0.0219 (D3H2LN); **p = 0.004 (MDA-MB-157); ***p = 0.00031 (MMTV-Myc), unpaired *t*-test.

We have previously shown that interference with the protein interactions of PAH2 domain of Sin3A protein by stable expression of SID peptide in MDA-MB-231 cells, induced expression of *RARβ2* [4]. Consistent with this, short term treatments of MDA-MB-231 cells with 31-mer SID peptide [6] increased the expression of *RARβ2* by 1.5-fold after 24 h, 8.6-fold after 72 h and 24-fold after 144 h of treatments (Figure 1A). A significant increase in *RARβ2* expression was also observed in mouse TNBC cells, 4T1, treated with SID peptide (Supplementary Figure S1). In addition to this, in a microarray experiment in MDA-MB-231 cells treated with SID peptide [6], pathway analysis revealed down-regulation of several genes like STRA6, DUSP1, HOXA3 and EGR1 that are known RAR γ target genes (Supplementary Table S1). To further test the effect of SID peptide on retinoid function, we quantified the endogenous levels of retinoids in two TNBC cell lines (MDA-MB-231 and 4T1) treated with SID peptide. LC-MS/MS was performed to measure total retinoic acid (RA) production in the presence of 2 μM RBP4-retinol with or without SID treatment. In comparison to cells treated with scrambled peptide control (Scr), SID treatment induced 50% increase in RA production in human MDA-MB-231 and 21% increase in mouse 4T1 cells (Figure 1B). For quantification of the neutral retinoids like retinol (ROL) and retinyl ester (RE), HPLC-UV was used. Compared to Scr-treated cells, there were no significant changes in ROL or RE in cells treated with SID peptide (Supplementary Figure S2A). Consistent with these results we have previously reported a SID-induced increase in expression of a retinoid target gene, CRBP1 [4], which is known to increase RA biosynthesis [21].

To test if SID-induced increase in RA also leads to activation of RAR-regulated transcription, three TNBC cells lines (D3H2LN, a MDA-MB-231 variant; MDA-MB-157 and MMTV-Myc) were transiently transfected with retinoic acid response element (RARE) driven GFP plasmid and then treated with SID or control scrambled (Scr) peptide. Mean fluorescence intensity of GFP was used to measure the activation of RARE-driven GFP expression (Figure 1C). In all the three cell lines tested, in comparison to the control, significant increase was observed in GFP expression upon SID treatment (41% increase in D3H2LN, 78% in MDA-MB-157 and 75% in MMTV-Myc; Figure 1C). Together our results demonstrate that SID peptide can activate retinoid signaling in TNBC cells.

### C16, a small molecule inhibitor mimetic of SID peptide, increases expression of RAR*β2* and, in combination with RARα agonist AM80, enhances retinoid signaling

The long-term goal of our laboratory is to translate the novel findings on SID function into treatment for patients with TNBC. For that purpose, we screened and reported [7] that Avermectins are SID peptide mimetics. Although, potent, these compounds are not readily soluble. Hence, we conducted structure-guided computational screen to identify compounds that are more soluble and hence better suited for studying the anti-tumor effects of SID decoys *in vivo*. An extensive structure-activity relationship (SAR) study of 115,000 small molecule compounds was conducted as reported previously [7], to identify small molecule inhibitors (SMIs) with enhanced and selective binding to the target mSin3A PAH2 domain. Amongst the initial group of candidate SMIs, we identified compound 16 (C16; IUPAC name: 4-(2,3-Dichlorophenyl)-3a,4,5,9b-tetrahydro-3H-cyclopenta[c]quinoline-6-carboxylic acid) that exhibited binding affinity Ki of 23.6 μM in fluorescence anisotropy competition assay (Supplementary Figure S3A). A detailed NMR titration (as previously described (Kwon et al., 2015)), established that C16 interacts with PAH2 through residues critical for the interaction between SIN3A PAH2 and the MAD SID domain (Supplementary Figure S3B). Consistent with its function as a PAH2 blocker, proximity ligation assay also confirmed that C16 disrupts the interaction between Sin3A and MAD1 (Supplementary Figure S3C). Similar to previous reports for SID decoys, treating MDA-MB-231 cells with C16 for 72 h significantly increased the expressions of *CDH1* and *ESR1* (Supplementary Figure S3D).

**Figure 2:**
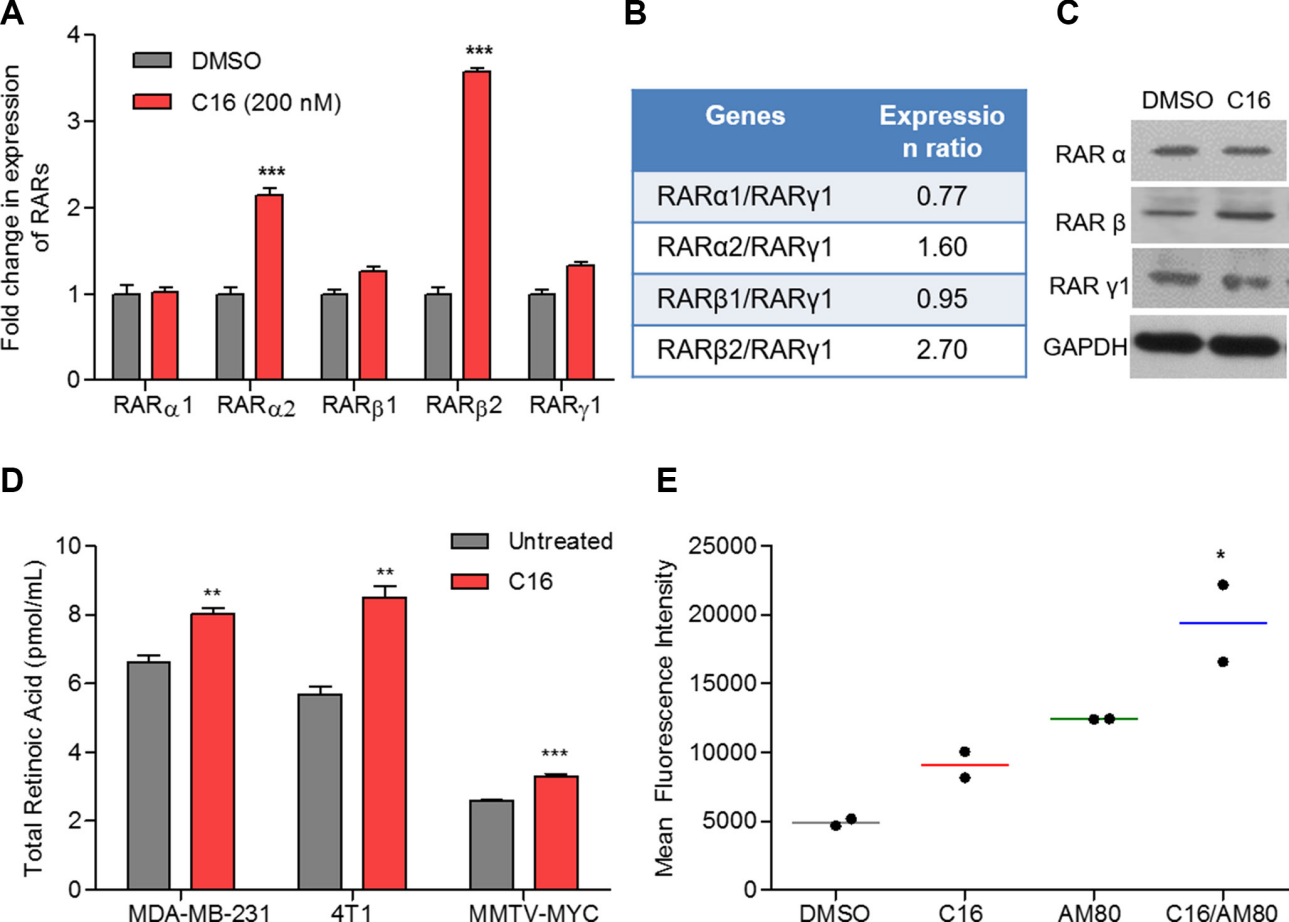
C16 modulates expression of RARs and enhances the retinoid signaling in combination with AM80 (**A**) qRT PCR for expression of *RARα1/2, RARβ1/2* and *RARγ1* in MDA-MB-231 cells treated with 200 nM C16 for 96 h. Error bars represent mean ± SD (*n* = 3). DMSO versus C16, ***p < 0.0001, unpaired *t*-test. (**B**) Ratio of relative expression of RARs measured in (A). (**C**) Western Blot for RARα, RARβ and RARγ1 in MDA-MB-231 cells treated with C16 at 200 nM for 96 h. (**D**) Endogenous retinoic acid levels in three breast cancer cell lines (MDA-MB-231, 4T1 and MMTV-Myc) treated with 200 nM C16 for 6 days. Error bars represent mean ± SD (*n* = 3). DMSO versus C16, **p = 0.0059 (MDA-MB-231); *p* = 0.0022 (4T1), *p* = 0.008 (MMTV-Myc), unpaired *t*-test. (**E**) Expression of RARE-driven GFP reporter in MDA-MB-231 cells treated with 200 nM C16 and/or 200 nM AM80 (*n* = 2). DMSO versus C16-AM80, *p < 0.05, one-way ANOVA.

Treatment of MDA-MB-231 cells with 200 nM C16, resulted in significant increase in expression of *RARα2* (2.1-fold) and *β2* (3.6-fold) mRNA (Figure 2A). Moreover, there was an increase in the ratio of RARα2/RARγ1 (1.6-fold over the untreated) and RARβ2/RARγ1 (2.7-fold) expressions (Figure 2B), suggesting possible activation of RARα/β specific pathways. At the protein level, significant increase was observed in RARβ, a RARα target gene (Figure 2C). No significant increase in the total RARα protein was observed in C16-treated cells. Differences in protein levels of specific RARα isoforms could not be verified due to lack of isoform-targeted antibodies. Similar to the SID peptide, C16 also induced 24% increase in RA production in MDA-MB-231 cells while in 4T1 and MMTV-Myc cells 50% and 27% increases were observed (Figure 2D). No change in levels of ROL or RE was observed (Supplementary Figure S2B). Further, in the presence of an RARα-specific antagonist, RO41-5253, C16 treatments neither activated the RARE-GFP reporter (Supplementary Figure S4A) nor increased *RARβ2* expression (Supplementary Figure S4B), suggesting C16-induced increase in RAR signaling is RARα-dependent.

To test the consequence of the C16-effect on RA production we examined the activation of a RARE-driven gene promoter in cells treated with C16. We also tested whether the RARE-driven transactivation is enhanced by a RARα specific agonist AM80; a drug approved for treatment of ATRA resistant APL in Japan [22–24]. MDA-MB-231 cells were transiently transfected with RARE-driven GFP reporter plasmid and treated with C16 and AM80, either alone or in combination. C16 treatments increased the expression of the RARE-GFP reporter by 84% (Figure 2E). Addition of AM80, increased the reporter activity to 300% (Figure 2E). Taken together our results clearly demonstrate the ability of a SID decoy, in combination with retinoids like AM80, to increase ligand-mediated activation of RAR signaling.

### C16 in combination with AM80 induces acinar morphogenesis and inhibits tumorsphere formation

**Figure 3:**
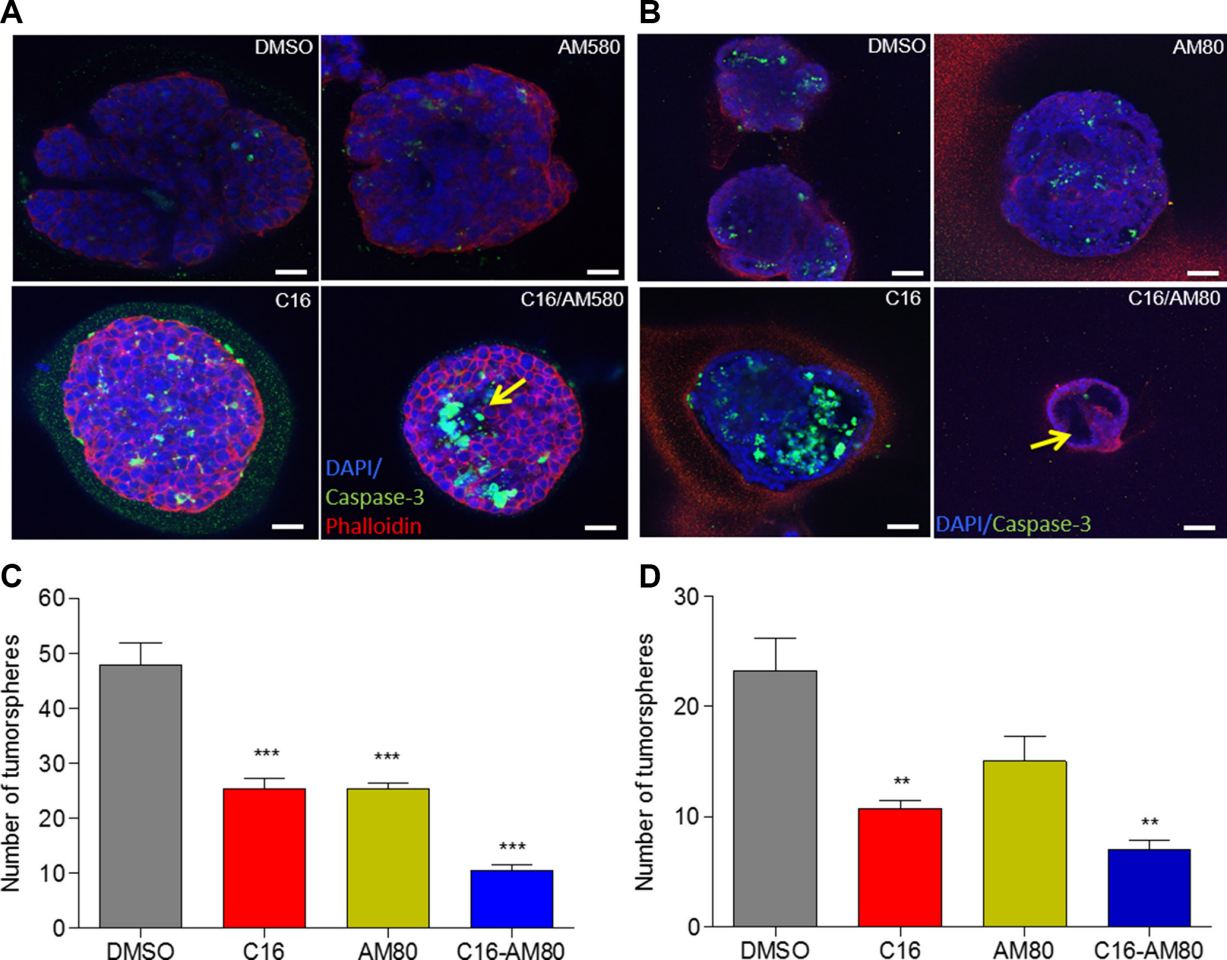
C16 in combination with AM80 induces morphogenesis and inhibits tumorsphere formation (**A**) Colony morphogenesis of 4T1 cells cultured in 3D Matrigel with 200 nM C16 and or 200 nM AM580 for 10 d followed by staining with DAPI (blue), caspase-3 (green) and phalloidin (red). Scale bar = 25 μm. Arrows indicate the partial acini formation. (**B**) Colony morphogenesis of 4T1 cells cultured in 3D matrigel with 200 nM C16 and/or 200 nM AM80 for 10 d followed by staining of the colonies with DAPI (blue) and caspase-3 (green). Scale bar = 50 μm. Arrows indicate the partial acini formation. (**C**) Tumorsphere assay of 4T1 cells treated with 200 nM C16 and/or 200 nM of AM80 for 7 d. Results show numbers of tumorspheres. Error bars represent mean ± SD (*n* = 3). DMSO versus C16 or AM80 or C16-AM80, ***p < 0.001, unpaired *t*-test. (**D**) Tumorsphere assay in MMTV-Myc cells treated with 200 nM C16 and/or 200 nM of AM80 for 7 d. Results show numbers of tumorspheres. Error bars represent mean ± SD (*n* = 3). DMSO versus C16, **p = 0.0064; DMSO versus C16-AM80, **p = 0.0019, unpaired *t*-test.

We have previously reported that SID decoys can induce morphogenesis and cellular differentiation in 3D cultures of TNBC cells in basement membrane matrix [4, 6]. The fact that our prior work linked RARα activation pathway to differentiation and cell death [25] and that we show here (Figure 2) ligand mediated activation of RARs, compelled further study of the effect of C16 in combination with RARα agonists on colony morphogenesis. We used two RARα specific agonists, AM80 and AM580. 4T1 cells cultured in Matrigel were treated with C16 alone, AM580, AM80 or the combination of C16 with each of the agonists. C16 treated colonies showed increased level of activated caspase-3 (Figure 3A and 3B), a precursor to cavitation and acini formation [4]. Treatment with the combination of C16 and AM580 further increased the levels of caspase-3 with evidence of rudimentary acini formation (Figure 3A). These effects were more profound with C16/AM80 combination in which the colonies were small, non-invasive with features resembling normal acinar morphogenesis (Figure 3B), suggesting that C16/RARα agonist combination can induce differentiation of TNBC cells cultured in matrix.

We next tested the effect of C16 in combination with AM80, on tumorsphere formation. Tumorspheres were generated by growing mouse TNBC cell lines 4T1 and MMTV-Myc in suspension cultures. These cultures were treated with DMSO (vehicle control), C16, AM80 or the combination of the two. Compared to DMSO, C16 and AM80 individually reduced the number of 4T1 tumorspheres by 47% and the combination of C16 with AM80 by 80% (Figure 3C). In MMTV-Myc cells, treatments with either C16 or AM80 resulted in 54% and 35% decrease in tumorsphere numbers, respectively, while the C16-AM80 combination decreased the number by 70% (Figure 3D).

### C16 prevents development of Myc-driven mammary hyperplasia

**Figure 4:**
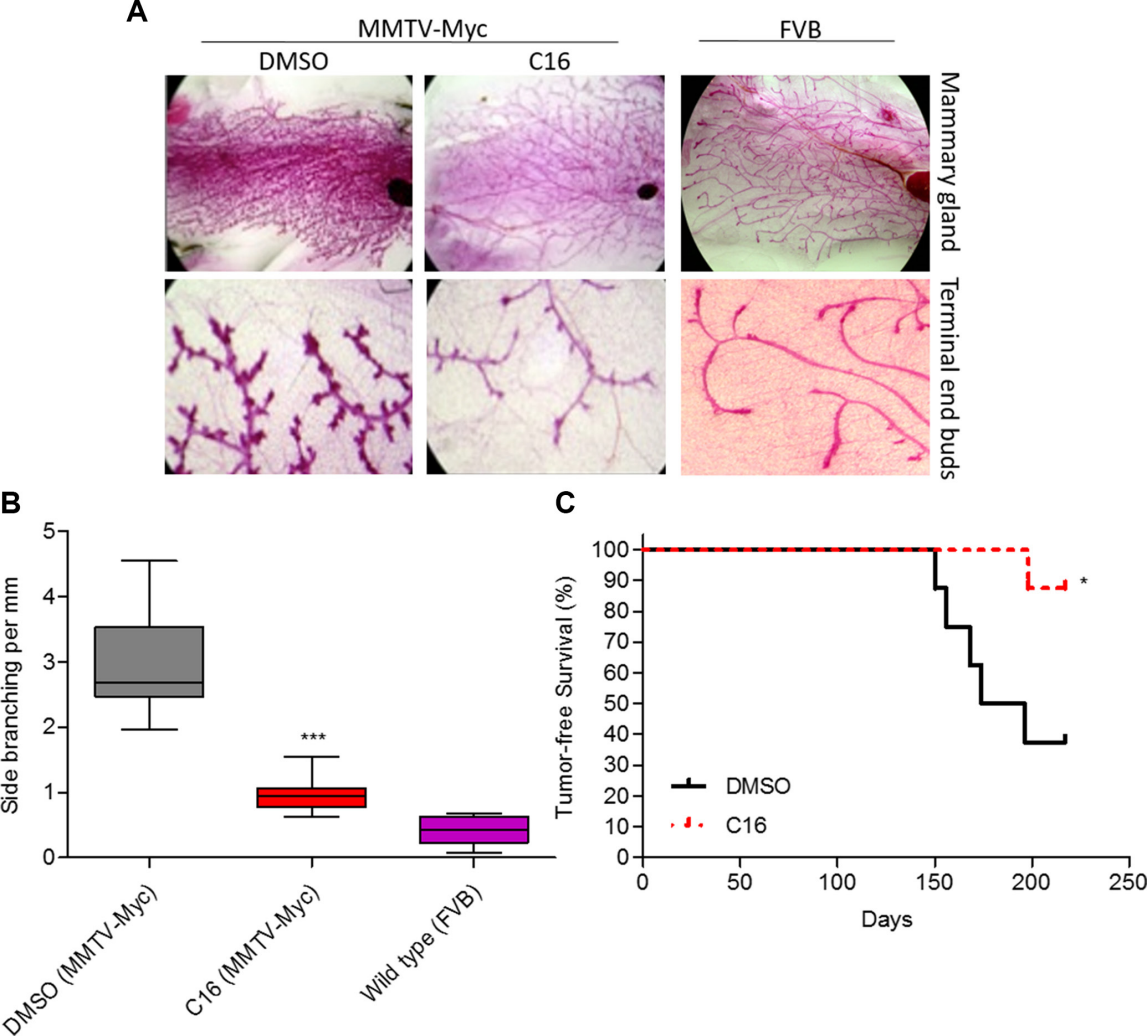
C16 prevents development of Myc-driven mammary hyperplasia (**A**) Representative images of the mammary gland architecture of 30 weeks old virgin MMTV-Myc oncomice (*n* = 16/group) treated with DMSO or C16 for 20 weeks. The far right panel shows an age-matched control of mammary gland isolated from healthy virgin FVB mouse. (**B**) Graph showing the number of side branches per mm of the mammary glands (*n* = 32) isolated in (A). DMSO versus C16, ***p < 0.0001, one-way ANOVA. (**C**) Kaplan-Meier plot showing tumor-free survival of MMTV-Myc oncomice treated with DMSO or C16 (*n* = 8/group). DMSO versus C16, *p = 0.0272, p, logrank test.

Although, testing of drug effects in colonies in matrix or in tumorspheres are accepted *in vitro* correlates of *in vivo* effects, current standards require that preclinical studies be conducted in animal models. Hence we tested the anti-tumor effect of C16 in MMTV-Myc oncomice – a model for TNBC [26, 27]. Since C16 as a single agent induced partial acinar morphogenesis and reduced the number of tumorspheres (Figure 3A–3D), we hypothesized that this effect might translate into induction of a more normal-like mammary tree in MMTV-Myc model *in vivo*. MMTV-Myc female oncomice present signs of mammary gland hyperplasia at ~10 weeks of age, which eventually progresses to ductal carcinoma *in situ* (DCIS) and to frank tumors between weeks 16 and 32 [28]. The hyperplastic phenotype is the result of c-Myc-driven anomalous expansion of the mammary stem cells [27] and the mammary stem cells are believed to be the targets of malignant transformation [29]. The stem cell expansion is believed to be the driver of the increased mammary ductal tree side branching [30]. Ten week old virgin MMTV-Myc mice were treated with C16 for 20 weeks, the mammary glands removed and the side branching of the mammary ductal tree was quantified as described previously [31]. Treatment with C16 changed the mammary gland morphology such that it resembled the mammary gland of a wild type FVB/N virgin or involuted mammary ductal tree (Figure 4A). This treatment reduced the density of the side branches (ductal tree hyperplasia) by ~3-fold (Figure 4B). Because C16 alone significantly reduced tumorsphere generation (Figure 3D) we examined whether it will also reduce primary tumor development and increase survival. A small cohort (*n* = 8 per experimental group) of MMTV-Myc females was treated with C16 (or DMSO as control) and monitored for appearance of palpable tumors. Of the 8 DMSO-treated mice 5 developed tumors in week 21 while only one of the eight C16 treated females developed a tumor on week 28.5. Kaplan Meyer analysis showed that treatment with C16 alone significantly increased tumor-free survival (Figure 4C).

### Treatment with C16-RARα agonist inhibits metastases and increases disease-free survival

We next asked whether the ability of C16 in combination with a RARα agonist to induce differentiation of TNBC cells, to block tumorsphere formation, and to inhibit mammary ductal tree hyperplasia, will translate into anti-tumor and anti-metastatic effect and will increase survival. To answer these important questions more broadly, the experiments were carried out in two TNBC mouse models; the well-established TNBC model of mouse 4T1 cells, which grow rapidly and closely mimic tumor growth and metastatic spread of stage IV human breast cancer, and the MMTV-Myc oncomice model. The experiments were designed to either administer treatment while the primary tumor was present and growing (neo-adjuvant therapy), or after the primary tumor was removed (adjuvant therapy). Because in the first setup, there is presumably continuous dissemination of tumor cells, inhibition of metastases would suggest that both dissemination and metastatic growth might be affected. In the second setup mainly the ability to block metastatic growth is being tested. To generalize our findings, we tested two RARα-specific agonists, AM80 and AM580, with slightly different structures and different potency in some assays [32].

**Figure 5:**
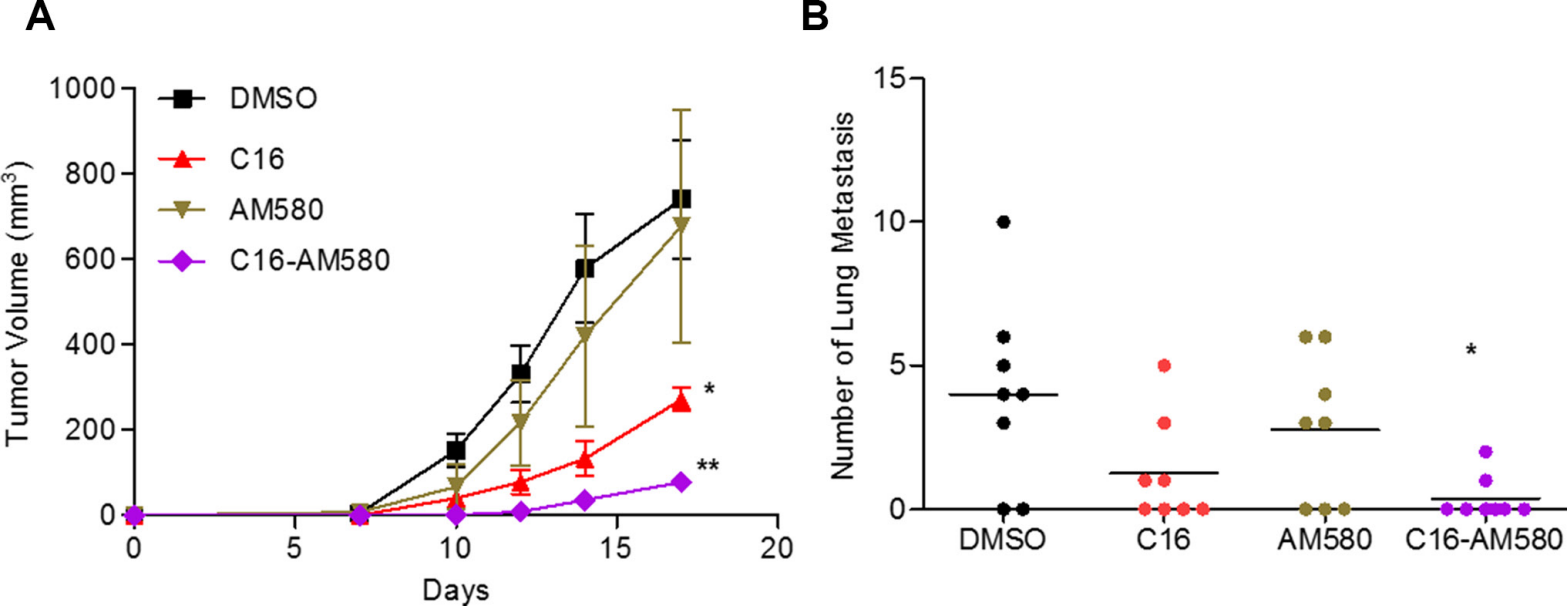
C16 in combination with AM580 inhibits primary tumor growth and lung metastases (**A**) Tumor progression in Balb/c mice (*n* = 8) inoculated with 4T1 cells and then treated with DMSO or C16 or AM580 alone or in combination for 17 days, and tumor volume quantified at the indicated times. DMSO versus C16, *p = 0.0273; DMSO versus C16-AM580, **p = 0.0032, unpaired *t*-test. (**B**) Lungs from sacrificed animals (A) were isolated and metastatic foci counted. DMSO vs C16-AM580, *p = 0.0158, Mann-Whitney test.

Balb/c mice bearing fast growing and rapidly metastasizing 4T1 tumors were treated with C16 or AM580 or C16 in combination with AM580. Treatment for 17 days with C16 alone (but not with AM580 alone) reduced the mean tumor volume by 63%. The combination reduced tumor volume by 90% (Figure 5A). Lung metastases was significantly decreased with C16 alone (median = 0.5 vs 4 for DMSO) and appeared to be significantly blocked in mice treated with the C16/AM580 combination as no macroscopic lung metastases was visible (Figure 5B). To test whether these findings extend to another model of TNBC, MMTV-Myc female mice bearing palpable tumors were treated with the combination of C16 and AM80 for 36 days, or with DMSO as control, and the tumor growth was monitored. As compared to the DMSO-treated group, C16/AM80 treatment reduced the tumor volume by ~36% (Supplementary Figure S5A). Importantly, in spite of the presence of a progressively growing tumor, this treatment reduced lung metastases by 90% (Supplementary Figure S5B), suggesting that dissemination, or growth of metastases or both are being inhibited by the C16/AM80 treatment.

**Figure 6:**
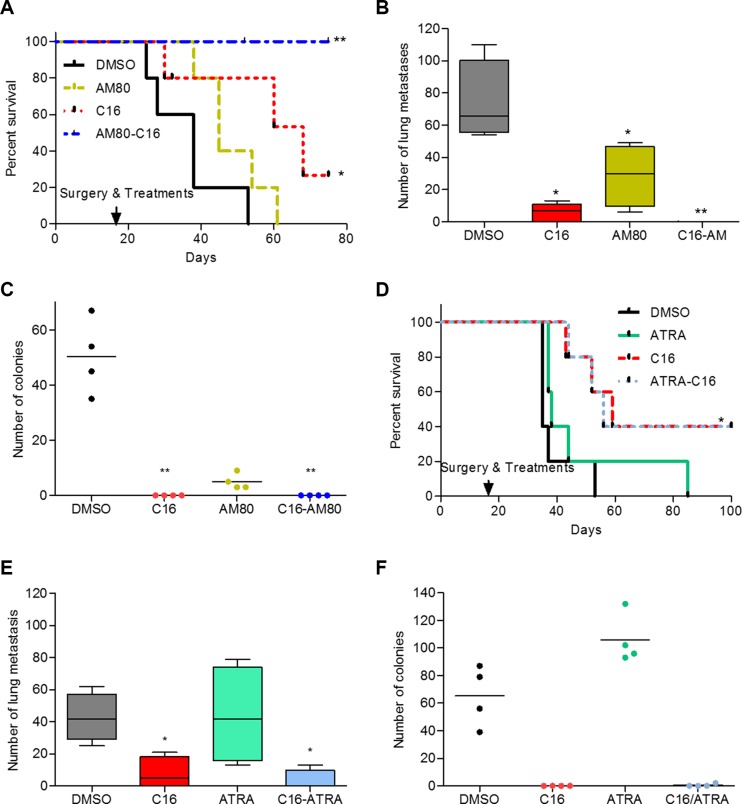
Adjuvant treatment with C16-AM80 combination inhibits metastatic dissemination and increases disease-free survival (**A**) Kaplan-Meier analysis showing disease-free survival following removal of primary tumors in Balb/c mice (*n* = 5) inoculated with 4T1 cells and treated with C16 and AM80 alone or in combination. DMSO versus C16, *p = 0.0343; DMSO versus C16-AM80, **p = 0.0025, logrank test. (**B**) Lungs from sacrificed Balb/c mice inoculated as described in (A) were isolated and metastases counted. DMSO versus, C16, *p = 0.0159, Mann-Whitney test; DMSO versus AM80, *p = 0.0286, Mann-Whitney test; DMSO versus C16-AM80, **p = 0.0039, one-way ANOVA (Mann-Whitney could not be applied due to repeated values of zero for C16-AM80 treated mice). (**C**) Quantification of the disseminated 4T1 tumor cells isolated from the bone marrow of sacrificed animals from (A). DMSO versus, C16, **p < 0.01; DMSO versus C16-AM80, **p < 0.01, one-way ANOVA. (**D**) Kaplan-Meier analysis showing disease-free survival following removal of primary tumors in Balb/c mice inoculated with 4T1 cells and treated with C16 and atRA alone or in combination. DMSO versus C16, *p = 0.02345, logrank test. (**E**) Lungs from sacrificed Balb/c mice inoculated as described in (D) were isolated and quantified for the number of metastases observed. DMSO versus C16 or C16-atRA, *p = 0.0286, Mann-Whitney test. (**F**) Quantification of the disseminated 4T1 tumor cells isolated from the bone marrow of sacrificed animals from (D).

The benefit of C16/AM80 combination treatment in the post-surgical adjuvant setting was also evaluated. This design assumes that if residual disease exists, it is present in the form of disseminated cells, which most likely also include cancer stem cells. To achieve this, the primary tumors were dissected and only then the treatment commenced. Following the resection of primary tumors (~300 mm^3^ at day 17 post tumor cell inoculation), developed by inoculating 4T1 cells in Balb/c mice, the animals were treated with DMSO, C16, AM80 and C16/AM80 once every day and followed for signs of cachexia as a sign of disseminated disease. All animals in DMSO-treated group showed symptoms of cachexia and were euthanized between day 8 and 33 post primary tumor resection with a median survival of 38 days (Figure 6A). In the C16-treated group, symptoms of cachexia appeared in three mice on day 10, 40 and 48 post-surgery while two mice did not show any clinical symptoms and were electively sacrificed at the end of the experiment (Figure 6A). In the AM80 group, following cachexia, the animals were euthanized between days 18 to 41 post-surgery and the median survival was 45 days (Figure 6A). The strongest effect was obtained with C16/AM80 adjuvant treatment wherein all mice (but one which died of cancer unrelated cause) had no symptoms of clinical disease or toxicity and were electively sacrificed on day 80 of treatment (Figure 6A). Following euthanasia, lungs were excised from each animal and examined for metastases. Compared to DMSO, treatment with either C16 or AM80 decreased lung metastases by 90% and 54% respectively (median values, C16 = 7; AM80 = 30 vs 65.5 for DMSO). Remarkably, no evidence of macroscopic lung metastases was detected in lungs isolated from mice receiving C16-AM80 combination treatment (Figure 6B). Whether the blocking of metastases is achieved through blocking of cancer stem cells (CSCs) (as would be suggested by the *in vitro* effects) remains to be determined. In parallel, bone marrow aspirates from both femurs of each mouse were cultured in a colony assay to quantify disseminated tumor cells (DTCs). C16 either alone, or in combination with AM80, successfully eradicated bone marrow DTCs. Interestingly, AM80 even as a single agent resulted in a significant 92% reduction in bone marrow DTCs (Figure 6C). Similar experiment was performed using atRA instead of AM80. In contrast to AM80, atRA either as a single agent or in combination with C16 did not decrease lung metastases, DTCs or improve overall rate of survival compared to C16 alone, as measured by Kaplan Meir plot (Figure 6D–6F). The effect of C16 or AM80 as single agents, or as a combination, on metastases (after primary tumor resection) was also tested in MMTV-Myc mice. Under this setup, all 3 treatments, C16 alone, AM80 alone and the combination of the two produced a significant reduction in lung metastases (Supplementary Figure S5C).

## DISCUSSION

We show here that by using the combination of SID decoys and RARα agonist it is possible to help “cure” mice of a TNBC-like tumor. This treatment works in a neoadjuvant and adjuvant setting, it eliminates disseminated cells in the bone marrow, blocks metastases and extends survival to possibly achieve a normal life span. Patients with TNBC tumors frequently fail standard chemotherapy, and although recent attempts at treatment with PARP inhibitors, PI3K inhibitors and modulators of the p53 family members have shown some early promise [33], no clinically validated drug target exist that are effective as neo-adjuvant or adjuvant therapy and frequently the tumor relapses locally, regionally or as systemic metastases.

Our approach was to restructure the epigenome by disrupting protein interactions between Sin3A PAH2 domain and a set of SID-containing chromatin-associated factors like MAD1 and PF1, [4, 6, 7], to promote differentiation and inhibit EMT and CSCs. We now show that SID decoys, and especially a small molecule C16 has a potent anti-tumor activity even as a single agent (Figures 4–6) to block mammary hyperplasia, inhibit tumor growth, tumor cells' dissemination, metastases and prolong tumor-free survival (Figures 4–6). In spite of these impressive single agent results in preclinical models, clinical experience teaches that even targeted monotherapy is seldom successful in long term; so we searched for combination therapy which will target TNBC vulnerabilities. Our search revealed that treatment of TNBC cells with C16 induces the expression of functional RARs, specifically RARα2 and RARβ2 and sensitized TNBC cells to retinoids (Figures 1–3). Our study is consistent with the use of LSD1 inhibitors to alter the epigenome and reactivate atRA-induced differentiation in AML [34]. The normal function of retinoids to induce differentiation is frequently altered in cancer, and targeting the retinoid-dependent pathway and, possibly, the cancer stem cells is an attractive anti-cancer strategy [11, 15, 35, 36].

Although atRA treatment was shown to achieve complete remission in APL, its success has not been reproduced in other tumors, including metastatic breast cancer [37]. There are several reasons for the loss of retinoid responsiveness in tumors. First, well supported by published evidence, is attributed to abnormal recruitment of epigenetic enzymes (the HDAC–containing corepressor complexes like Sin3A-HDAC complex and DNMTs) that silences the retinoid-response genes such as RARα and RARβ2 [15, 17–20]. Accordingly, it was possible to restore RARα and RARβ2 expressions in cells treated with HDAC inhibitors (TSA) or DNMT inhibitor (azacitidine) and then inhibit cell proliferation by retinoid treatment [38, 39].

However, other considerations limit the use of retinoids beyond APL. First atRA used to treat APL activates all the RARs, including RARγ1, which was shown to activate pro-oncogenic signals and CSC proliferation in breast cancer [22, 40]. We have previously reported an imbalance in RARα/RARγ expression could be reversed by treatment with RARα agonist and activation of RARβ2 in Myc-driven TNBC [25]. In fact, activation of RARα has been shown to be sufficient for achieving retinoid response in both ER+ and ER–breast cancer cells [41] while atRA, by activating PPAR β/δ, induced tumorigenic effects [42, 43]. This becomes especially important for ER–cells (like TNBC) that express higher levels of PPAR β/δ than ER+ cells [15]. These considerations and the fact that the synthetic retinoid AM80 (not atRA) is resistant to degradation by stromal retinoid metabolizing enzymes CYP26A1 [41, 44] and therefore expected to sustain higher plasma levels with stronger anti-tumor activity, prompted us to consider it as a partner drug for the SID decoys [24]. In agreement with this, a remarkable difference was observed in anti-tumor activity of AM80 versus atRA (Figure 6). The marked increase in disease-free survival without measurable toxicity observed in mice treated with C16-AM80 was associated with eradication of disseminated tumor cells. Moreover, the ability of C16 to inhibit mammary gland hyperplasia further motivates investigations for its use in chemoprevention.

The precise mechanism of action via which SID decoys activate retinoid response is not clear. It is debatable if the retinoid-target genes are also the primary transcription targets of Sin3A; although earlier studies have shown protein-protein interactions between Sin3A and corepressors like NCoR and SMRT that directly regulate the RAR-target promoters [45]. We predict that the anti-tumor effects of C16-AM80 are due to enhanced retinoid-regulated transcription activation although identification of other genes within this complex network warrant further investigations. Further, retinoid signaling regulates mammary epithelial cell growth and differentiation via activation of both retinoic acid (RA) and retinoid X receptors (RXRs) [15]. Studies that venture into the contributions of RXRs in the observed phenotype of C16 will be critical; especially because of the known interaction between RXRα and the PAH2-interacting transcription factor TGIF1 that is known to inhibit retinoid signaling [46, 47]. However, we also cannot rule out the possibility that the enhanced sensitivity of C16-treated TNBC cells to retinoids could be an indirect effect of Sin3A functions in regulation of pathways that crosstalk with retinoid signaling like estrogen and Wnt signaling that have previously been shown to be affected by SID decoys [4, 6, 7]. Further, SID decoys favor transition from a basal to luminal phenotype [4] that is more retinoid sensitive [48]. Nonetheless, these data establish existence of a crosstalk between Sin3A and retinoid signaling and targeting Sin3A by C16 in combination with AM80 may be a promising adjuvant therapy for treating or preventing metastatic TNBC.

## MATERIALS AND METHODS

### Cell culture

The mouse metastatic mammary 4T1 tumor cell line (Cat# CRL-2539) and human MDA-MB-231 breast adenocarcinoma cell line (Cat# HTB-26) were purchased from the American Type Culture Collection (ATCC). The MDA-MB-231-Luc-D3H2LN Bioware (D3H2LN) cell line [49] was purchased from PerkinElmer (Cat# 119369). The mouse mammary tumor MMTV-Myc cell line has been previously reported [25, 50]. Cell lines were authenticated by short tandem repeat (STR) profiling in accordance with the standard ASN-0002-2011 in April 2015 and March 2016 (DDC Medical). 4T1 cells were maintained in RPMI supplemented with 10% fetal bovine serum (FBS) and 1% Antibiotic-Antimycotic solution (Invitrogen). The MDA-MB-231 cell line was maintained in DMEM supplemented with 10% FBS, 1% GlutaMAX (Invitrogen), 10 mM HEPES, 1 mM sodium pyruvate, non-essential amino acids and 1% antibiotic-antimycotic solution. MMTV-Myc cells were cultured in DMEM/F12 medium supplemented with 5% FBS, 1% GlutaMAX, 10 mM HEPES, and 1% antibiotic-antimycotic solution.

### Peptides and C16

MAD-SID peptide (SID: YGRKKRRQGGG-VRMNIQMLLEAADYLERRER), MAD scrambled peptide (Scr: YGRKKRRQGGGEQRARRIMERLLE YNMVADL) [6] were synthesized to a purity level of 95% as assessed by analytical reversed phase-high performance liquid chromatography (BioSynthesis, Inc. Lewisville, TX). C16 (IUPAC: 4-(2, 3-Dichlorophenyl)-3a, 4, 5, 9b-tetrahydro-3H-cyclopenta[c]quinoline-6-carboxylic acid) was initially screened and supplied by laboratory of Dr. Ming-Ming Zhou at Icahn School of Medicine at Mount Sinai, New York. Additional C16 was purchased from Mcule, Inc (Palo Alto, CA) and Ambinter (c/o Greenpharma, Orleans, France).

### Immunofluorescence

Cells were cultured on 8-well chambers (BD Biosciences) and fixed with 4% paraformaldehyde/PBS for 15 min at room temperature. For 3D cultures cells were seeded (3 × 10^3^/well) in quadruplicate onto Matrigel (BD Biosciences) beds in 8-well culture slides to prepare three-dimensional cultures as described earlier [61]. The media was changed every 48 h for 8 consecutive days. Colony morphology was determined by phase-contrast microscopy. For immunostaining, cells were permeabilized with 0.5% Triton X-100/PBS and blocked with 10% normal goat serum (Invitrogen) in PBS for 1 h. Primary antibodies were incubated overnight at 4°C in blocking buffer and washed 3 times with washing buffer (0.05% Triton X-100/PBS) and once with PBS. Secondary antibodies (dilution 1:200 in 1% normal goat serum/PBS) were added for 1 h and then washed. The samples were then mounted with ProLong Gold antifade reagent with DAPI (Molecular Probes/Invitrogen, CA), following the manufacturer instructions. All incubations and washes were done at 4 or 25°C as required. Confocal microscopy was performed using a Leica SP5 confocal microscope at the Shared Instrumentation facility of department of Hematology at Mount Sinai School of Medicine, NY.

### Identification of C16 as small molecule mimetic of SID

Computational screening of chemical compounds was conducted in the laboratory of Prof. Ming-Ming Zhou at Icahn School of Medicine at Mount Sinai, New York, as described previously in Kwon et al., 2015. Top scoring candidate SMIs were tested for their binding to the SIN3A PAH2 domain experimentally by NMR spectroscopy as previously described [7]. The binding affinity of C16 for SIN3A was assessed in a fluorescence anisotropy competition assay as described previously [6].

### Proximity ligation assay

MDA-MB-231 cells plated onto coverslips in 12 well plates with or without C16 treatment were stained with monoclonal SIN3A (sc-5299) 1:100 and polyclonal MAD1 (sc-222) 1:1000 following the Duolink protocol according to the manufacturer's instructions (Olink Bioscience) except utilizing 1% BSA in PBS as a blocking reagent and carrying out initial washes in PBS. Cells were counterstained in To-pro-3-iodide in PBS, 3 × 5 min washes at RT and mounted in Vectashield mounting medium (vector labs). Images were collected on a Zeiss LSM700 confocal microscope and the Duolink software was utilized to quantitate the signals.

### RARE reporter assay

TNBC cell lines described were treated with 2.5 μM SCR or SID, 200 nM C16, 200 nM AM80 for 96 h and transiently transfected with 4 μg of DNA of the RARE-EGFP reporter [51] to detect the activation of RAR-associated signaling by the increase in EGFP fluorescence by fluorescence microscopy and FACS analysis. All transfections were done using Turbofect (ThermoScientific) in accordance with the manufacturer's instructions. In experiments involving RARα antagonist, MDA-MB-231 cells were treated with 500 nM RO41-5253 (Sigma Aldrich) alone or in combination with C16 for 96 h followed by RARE-GFP reporter assay described above.

### Quantification of retinoic acid, retinyl esters and retinol

MDA-MB-231 and 4T1 cells were assayed for impact of SID peptide, and MDA-MB-231, 4T1 and MMTV-Myc cells were assayed for effect of C16 on retinoic acid (RA) production. One set of cells were treated with DMSO, 2.5 μM SCR or SID peptide, 200 nM C16 for 96 h. In a second set each treatment was combined with 2 mM RBP4-ROL for the last 48 h. Media was collected from cell cultures and cells were lysed. Media and cell lysates were frozen and kept at −80°C until extraction. Media and cell lysates from each condition were assayed separately for retinoid content. Retinoids standards were purchased from Sigma-Aldrich (St. Louis, MO, USA) and handled under yellow light. Media and cell lysates were extracted under yellow light using a 2-step liquid-liquid extraction, as described previously [52–54]. Retinoids were quantified in extracted samples within 1 day by liquid chromatography-tandem mass spectrometry (LC-MS/MS) for RA isomers using an AB Sciex 5500 QTRAP or by high-performance liquid chromatography with ultraviolet detection (HPLC-UV) for neutral retinoids (retinol, REs) using a Waters AQUITY UPLC [52–55]. Retinoid content was normalized per milliliter (media or cell lysate extracted).

### Tumorspheres assay

4T1 or MMTV-Myc cells (1 × 10^3^) were plated in ultra-low adhesion 6-well plates (Corning, Corning, NY) and incubated in serum-free F12/DMEM (Cellgro) supplemented with 20 ng/ml EGF, 0.5% Matrigel and 1:50 B27 Supplement (Invitrogen) for 3 days at 37°C in a humidified atmosphere of 5% CO2 and were then treated with DMSO, 200 nM C16, 200 nM AM80 or the combination of C16-AM80 for 7 days. The number of tumorspheres per well (triplicates) were counted.

### Quantitative real-time PCR

RNA was isolated using RNeasy Plus Mini Kit (Qiagen), and cDNA was prepared using Superscript First-Strand Synthesis System for RT-PCR Kit (Invitrogen) or iTaqScript (Bio-Rad), all following manufacturers' instructions. Quantitative real-time PCR was performed using manufacturers' instructions for QuantiTect SYBR Green PCR (Qiagen) or iTaq Universal SYBR Green Supermix (Bio-Rad) kits on Opticon or CFX96 machines (Bio-Rad) with annealing temperature 54°C with 50–250 ng cDNA and 6 pmols of gene specific primers (Supplementary Table S2 and [6]) per reaction. For determination of gene expression, the “delta-delta Ct method” was used relatively to RPL30 housekeeping genes.

### *In vivo* studies

#### Xenografts

Myc and 4T1 TNBC cells (5 × 10^4^ cells/mouse) were injected orthotopically in the mammary gland #4 or #9 in 8 weeks old FVB or BALB/c females respectively (*n* = 8 per experimental group). The FVB mice receiving the Myc cells were treated with 72 ug/kg/day (200 nM) C16 or DMSO (control). The BALB/c mice receiving the 4T1 cells were treated with DMSO, C16 (same dose used for the Myc mice), AM580 or AM80 (0.3 mg/kg/day) or the combination of C16 with either AM80 or AM580. All the treatment started 24 h after the inoculation of the cells. The mice were fed ad libitum. Tumor latency and growth was measured. Tumor volumes were calculated as ellipsoids (Dxd^2^/2) by measuring the main diameter (D) and the smaller diameter (d) and plotted vs. time (days). The experiment was stopped when tumors in the control group reached ~800 mm^3^. At the end of the experiments the lungs were stained with Bouin fixative solutions (Sigma) and the overt lung metastases counted. For experiments with post-surgery adjuvant treatments, primary tumors were surgically removed when the tumor volume was ~300 mm^3^. Twenty-four hours post-surgery, the mice were treated with above mentioned doses of DMSO, C16, AM80 as single agents or in combination. The mice were monitored for overall health and disease-free survival and euthanized when signs of cachexia were detected.

### Lung and bone marrow metastases dissemination studies

To determine the effect of the treatment described above on the metastatic potential, BALB/c mice were inoculated subcutaneous (s.c.) with 5 × 10^3^ 4T1 cells in the interscapular space, when the tumors reached 300 mm^3^ were surgically ressected under anesthesia/analgesia (ketamine/xylazine) following IACUC guidelines. The day after surgery the mice received the treatments, DMSO, AM80, C16 and C16/AM80 at the doses described. The mice were monitored for signs of cachexia (changes in weight, temperature, fur condition, activity, lethargy, respiratory distress), when signs of cachexia were detected the mice were euthanized and the lungs were fixed in Bouin fixative solution and overt lung metastases counted. Non-Parametric statistical analysis was used to determine the significance of the differences observed. For measuring the disseminated tumor cells aspirates were also collected from the bone marrow of femur, washed with PBS and selecting the 4T1 cells by culturing in the presence of selection marker thioguanine as described earlier [56].

### MMTV-Myc oncomice

To determine the effect of C16 on mammary ductal tree hyperplasia, DCIS and pre-neoplastic lesions, MMTV-Myc oncomice were used. Expression of c-Myc was genotyped by tail PCR of 10-weeks old virgin MMTV-Myc mice. The mice were then divided in 2 groups (*n* = 16/group) and treated everyday with either DMSO (0.01%) or C16 (72 μg/kg/day in 100 ul of PBS) for 20 weeks. At the end of the experiment the inguinal mammary glands (#4 and #8, a total of 32 glands per experimental group were analyzed) were removed and fixed for whole mounts staining with carmine alum as described previously [31, 40]. To determine the effect on tumor progression MMTV-Myc females (*n* = 8) bearing palpable tumors were treated, in this case, with the combination of C16 (72 μg/kg/day) and AM80 (0.2 mg/kg/day) for 35 weeks. Tumor growth was measured twice a week. Mice were euthanized when tumors reached 1 cm^3^ in volume following which overt lung metastases were counted and the inguinal glands that did not show palpable tumors were recovered for mammary ductal tree analysis by whole mounts as described above.

### Statistical analyses

Statistical analyses were performed with GraphPad Prism software (version 5.0). The experiments were conducted with at least three independent experiments unless otherwise mentioned. Where shown, *p* values were calculated using the unpaired Student's *t*-test, Mann-Whitney or one-way ANOVA as indicated.

### Study approval

All the *in vivo* work done with mice was done following the IACUC guidelines. Animal Welfare Assurance Number: A3111-01.

## SUPPLEMENTARY MATERIALS


