# Supplementary material for: Blocking the PAH2 domain of Sin3A inhibits tumorigenesis and confers retinoid sensitivity in triple negative breast cancer
Source: Oncotarget. 2016 Jun 7;7(28):43689–702. doi: 10.18632/oncotarget.9905 (PMC5190053; doi:10.18632/oncotarget.9905)
Supplement: Supplementary file 1 [file oncotarget-07-43689-s001.pdf]

Blocking the PAH2 domain of Sin3A inhibits tumorigenesis and confers retinoid sensitivity in triple negative breast cancer

Supplementary Materials

Supplementary Table S1: List of RARγ target genes downregulated in MDA-MB-231 cells treated with SID peptide compared to SCR treatment as identified by microarray analysis

Target	Expression change (Log Ratio)	Regulation by RARγ	Prediction for RARγ activity
STRA6	– 1.060	Upregulates	Inhibited
HOXA3	– 1.050	Upregulates	Inhibited
EGR1	– 1.050	Upregulates	Inhibited
DUSP1	– 1.380	Upregulates	Inhibited

Supplementary Table S2: List of primers used for qRT-PCRs

Gene	Sense primer	Antisense primer
RARA1	GGAACCCCATCGGCCCCCT	GGGGGCCATGTCCTGTGATGC
RARA2	AACCGGGCCTGTTTGCTCCC	AGCGGGGTGGAGTACGGACC
RARB1	ACAGGCTTTTAGCTGGCTTGTCT	TGCCCAGGACTCACTGACAGA
RARB2	GGGGGACCAGAATTCCCCCA	GATCGCTCGCGTTCTCGGCA
RARG1	TCCCACTCCAGCTACGGCCC	CTTCAGCCACAGCCCCTGCC

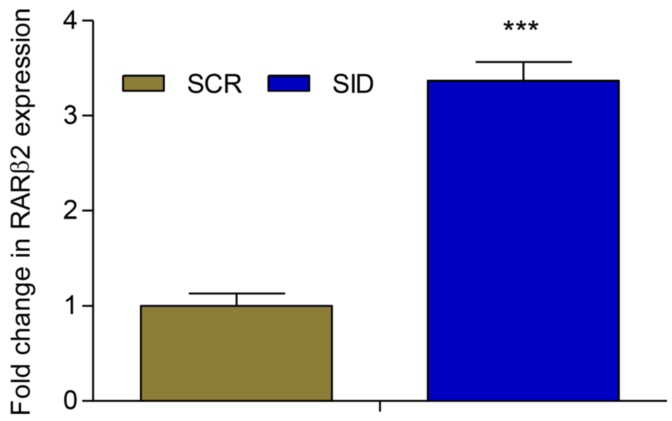

Supplementary Figure S1: SID peptide treatment increases RARβ2 expression in 4T1 cells. qRT PCR for expression of *RARβ2* in 4T1 cells treated with 2.5 μM SID peptide for 144 h. Error bars represent mean ± SD (n = 3). SCR vs SID, ***$p < 0.0001$, unpaired t-test.

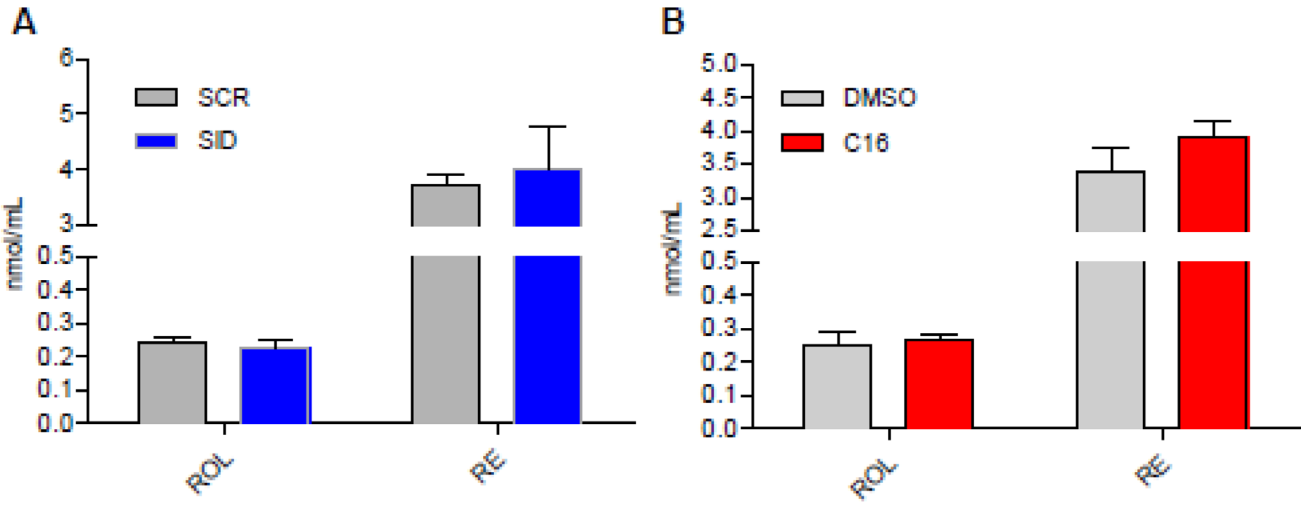

Supplementary Figure S2: SID decoys do not alter the endogenous levels of retinol and retinyl ester. (**A**) Quantification of retinol (ROL) and retinyl ester (RE) in MDA-MB-231 cells treated with scrambled (scr) and SID peptides. Error bars represent mean ± SD (n = 3). (**B**) Quantification of retinol (ROL) and retinyl ester (RE) in MDA-MB-231 cells treated with DMSO or C16. Error bars represent mean ± SD (n = 3).

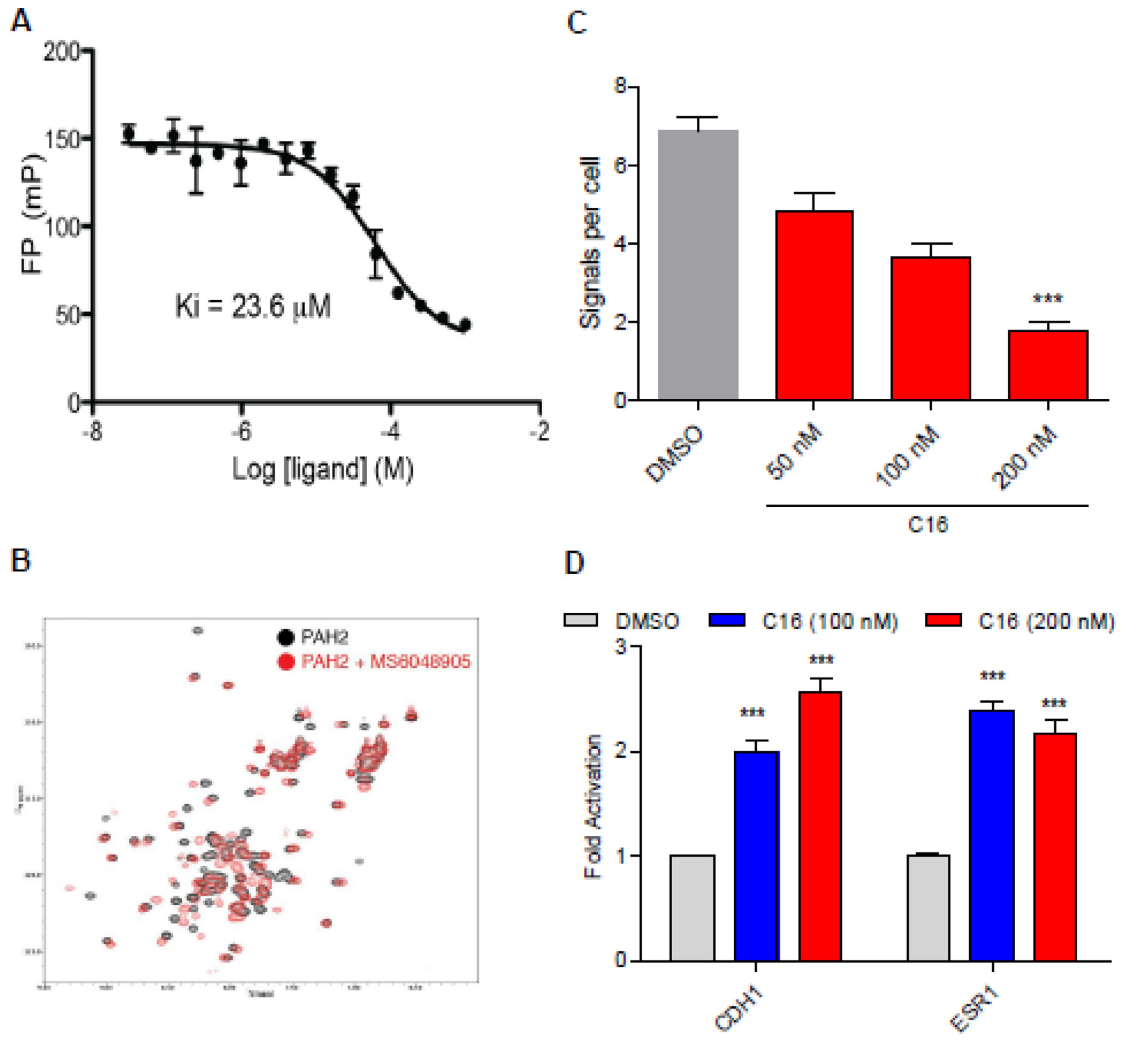

Supplementary Figure S3: Structure-guided discovery of C16 as a chemical ligand for the SIN3A PAH2 domain. (**A**) Binding affinity of C16 for the PAH2 domain of Sin3A determined by fluorescence polarization competition assay using a FITC-conjugated MAD peptide as a probe. (**B**) Two-dimensional ^{15}N-HSQC spectra of the ^{15}N-labeled SIN3A PAH2 domain (0.1 mmol/L) in the free form (black) and in the presence of C16 (MS6048905) (0.2 mmol/L; red). (**C**) Quantification of proximity ligation assay (PLA) analyzing the interaction between SIN3A and MAD1 in MDA-MB-231 cells treated with C16 (50 nM, 100 nM and 200 nM) for 72 h compared to the DMSO control. DMSO versus C16, ***$p < 0.001$, p, unpaired *t*-test. (**D**) qRT PCR for expression of *CDH1* and *ESR1* in MDA-MB-231 cells treated with 100 nM and 200 nM C16 for 72 h. DMSO versus C16, ***$p < 0.001$, unpaired *t*-test. Error bars represent mean ± SD ($n = 3$).

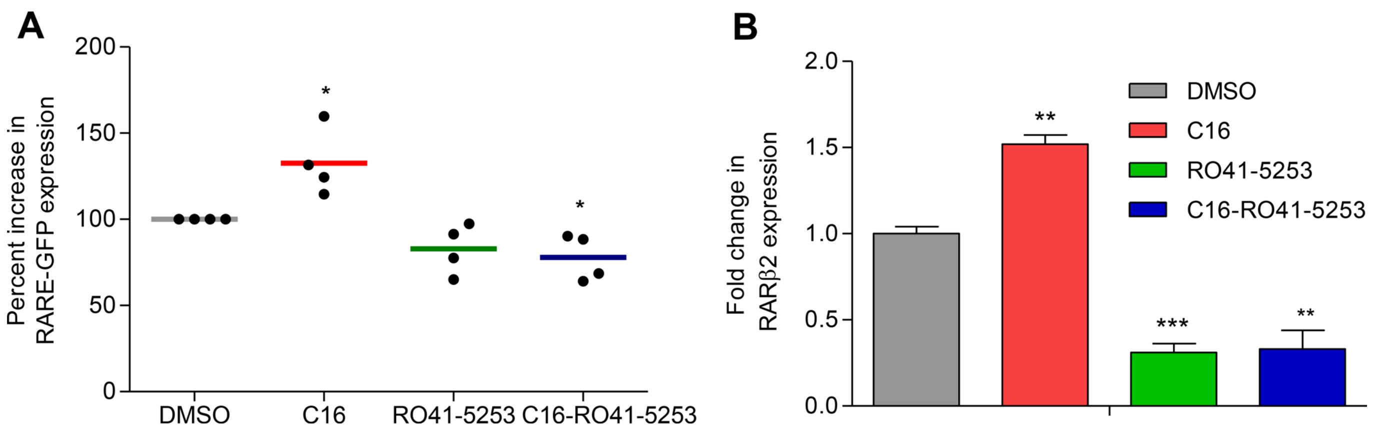

Supplementary Figure S4: Activation of retinoid signaling by C16 is RARα-dependent. (**A**) Expression of RARE- driven GFP reporter in MDA-MB-231 cells treated with 200 nM C16 and/or 500 nM RARα antagonist RO41-5253 for 96 h ($n = 4$). DMSO versus C16, *$p = 0.0440$; DMSO versus C16-RO-41-5253, *$p = 0.0462$, one sample *t*-test. (**B**) qRT PCR for expression of RARβ2 in MDA-MB-231 cells treated with 200 nM C16 and/or 500 nM RARα antagonist RO41-5253 for 168 h. Error bars represent mean ± SD ($n = 3$). DMSO versus C16, **$p = 0.0014$; DMSO versus RO41-5253, ***$p = 0.0005$; DMSO versus C16-RO41-5253, **$p = 0.0046$, unpaired *t*-test.

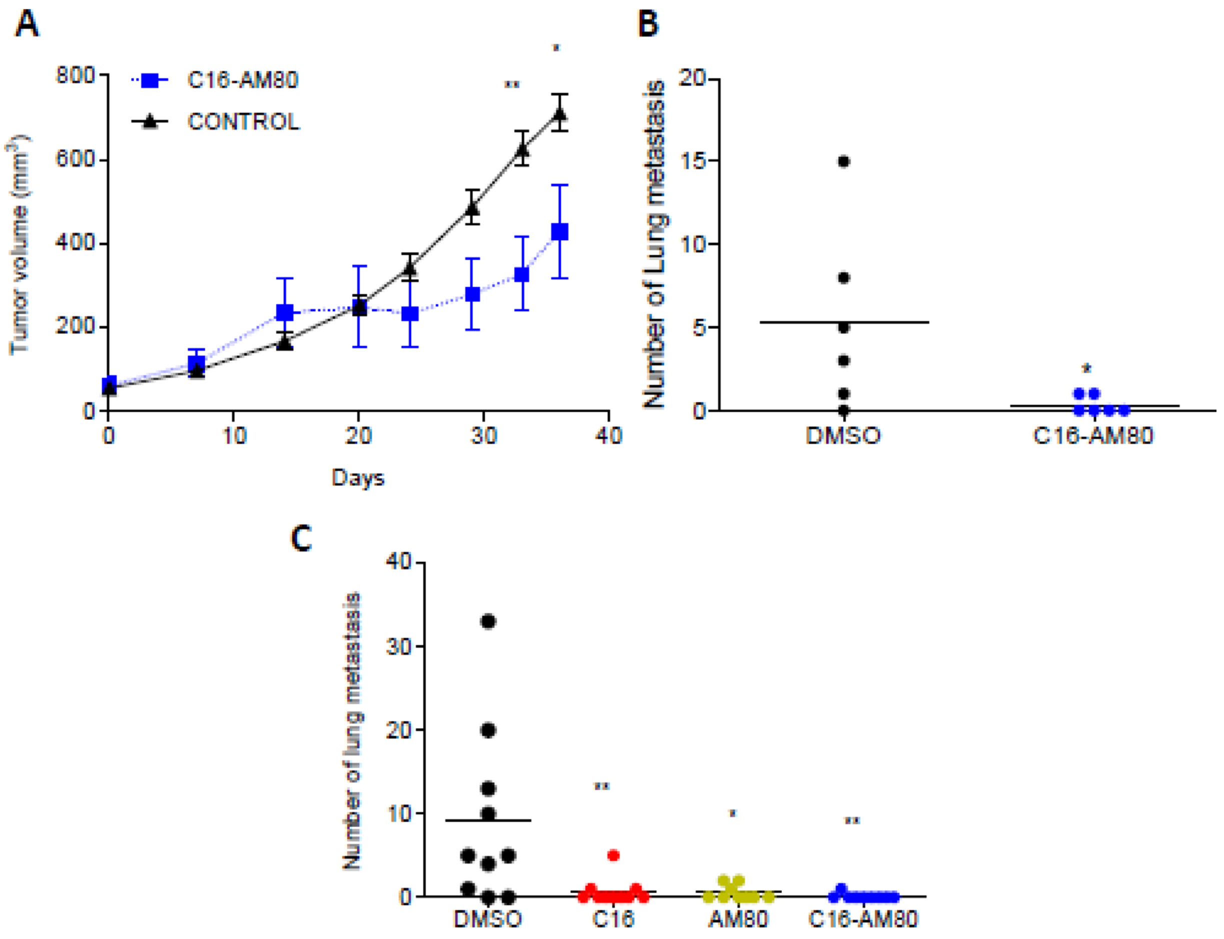

Supplementary Figure S5: C16-AM80 inhibits mammary hyperplasia, tumor progression and lung metastasis in MMTV-Myc oncomice. (**A**) Progression of spontaneous tumors formed in MMTV-Myc oncomice treated with DMSO or combination of C16 with AM80. Tumor volume was quantified at the indicated time points. DMSO versus C16-AM80, **$p < 0.01$ (day 33) and *$p < 0.05$ (day 36), one-way ANOVA. (**B**) Lungs from sacrificed animals (A) were isolated and quantified for the number of metastasis observed. DMSO versus C16-AM80, *$p = 0.041$, Mann Whitney test. (**C**) Number of lung metastases in FVB mice treated with C16 alone, AM80 alone or in combination after resection of primary MMTV-Myc tumor. DMSO versus C16, **$p < 0.01$, DMSO versus AM80, *$p < 0.05$, DMSO versus C16-AM80, **$p < 0.01$, one-way ANOVA.
